# Supplementary material for: Cost of Blood and Body Fluid Occupational Exposure Management in Beijing, China
Source: Int J Environ Res Public Health. 2020 Jun 12;17(12):4192. doi: 10.3390/ijerph17124192 (PMC7345934; doi:10.3390/ijerph17124192)
Supplement: Supplementary file 1 [file ijerph-17-04192-s001.pdf]

Supplementary Materials

Table S1. List of BBF exposure management regulations involving "safety injection" in China.

Year of enactment	Issuer	Title
2004	National Health and Family Planning Commission	Guiding Principles for the Work of HIV Professional Occupational Exposure Protection (Trial) [11]
2009	Ministry of Health	Guidelines for the Prevention of Occupational Exposure to Blood-borne Pathogens [12]
2015	National Health and Family Planning Commission	Occupational Exposure to HIV Treatment Procedures [13]
2015	Chinese Medical Association	Guidelines for the Prevention and Treatment of Chronic Hepatitis B (2015 Update) [14]
2015	Chinese Medical Association	Guide to Prevention and Treatment of Hepatitis C [15]

Table S2. Descriptions of direct cost items included from the survey.

Process	HBV	HCV	TP	HIV	Unknown
Laboratory tests	HBSAg<br>HBSAb<br>HBeAg<br>HBeAb<br>HBcAb	CBC<br>HCVAb<br>HCV-RNA	RPR<br>TPPA	HIV p24 antigen test<br>HIV antibody test<br>HBV, HCV, TP related tests	HBV tests<br>HCV tests<br>TP tests<br>HIV tests
Medical supplies	Blood collection tube<br>Injection set	Blood collection tube	Blood collection tube<br>Injection set	Blood collection tube<br>Injection set	Blood collection tube
PEP	Hepatitis B vaccine,<br>HbIg		Penicillin,<br>Erythromycin	Truvada, RAL<br>AZT, 3TC<br>HIVIG	

Note: HIV, human immunodeficiency virus; HBV, hepatitis B virus; HCV, hepatitis C virus; TP, *Treponema pallidum*; PEP, post-exposure prophylaxis; HBIg, hepatitis B hyper-immune globulin; HIVIG, anti-HIV immune globulin; RAL, raltegravir; AZT, azidothymidine; 3TC, lamivudine.
